# Feasibility of ultrasound radiomics based models for classification of liver fibrosis due to *Schistosoma japonicum* infection

**DOI:** 10.1371/journal.pntd.0012235

**Published:** 2024-06-13

**Authors:** Zhaoyu Guo, Miaomiao Zhao, Zhenhua Liu, Jinxin Zheng, Yanfeng Gong, Lulu Huang, Jingbo Xue, Xiaonong Zhou, Shizhu Li

**Affiliations:** 1 National Institute of Parasitic Diseases, Chinese Center for Disease Control and Prevention (Chinese Center for Tropical Diseases Research); National Key Laboratory of Intelligent Tracking and Forecasting for Infectious Diseases; NHC Key Laboratory of Parasite and Vector Biology; WHO Collaborating Centre for Tropical Diseases; National Center for International Research on Tropical Diseases, Shanghai, China; 2 Department of Ultrasound, The Yancheng Clinical College of Xuzhou Medical University, The First People’s Hospital of Yancheng, Yancheng, Jiangsu, China; 3 School of Global Health, Chinese Center for Tropical Diseases Research, Shanghai Jiao Tong University School of Medicine, Shanghai, China; 4 School of Public Health, Fudan University, Shanghai, China; Baylor College of Medicine, UNITED STATES

## Abstract

**Background:**

Schistosomiasis japonica represents a significant public health concern in South Asia. There is an urgent need to optimize existing schistosomiasis diagnostic techniques. This study aims to develop models for the different stages of liver fibrosis caused by *Schistosoma* infection utilizing ultrasound radiomics and machine learning techniques.

**Methods:**

From 2018 to 2022, we retrospectively collected data on 1,531 patients and 5,671 B-mode ultrasound images from the Second People’s Hospital of Duchang City, Jiangxi Province, China. The datasets were screened based on inclusion and exclusion criteria suitable for radiomics models. Liver fibrosis due to *Schistosoma* infection (LFSI) was categorized into four stages: grade 0, grade 1, grade 2, and grade 3. The data were divided into six binary classification problems, such as group 1 (grade 0 vs. grade 1) and group 2 (grade 0 vs. grade 2). Key radiomic features were extracted using Pyradiomics, the Mann-Whitney U test, and the Least Absolute Shrinkage and Selection Operator (LASSO). Machine learning models were constructed using Support Vector Machine (SVM), and the contribution of different features in the model was described by applying Shapley Additive Explanations (SHAP).

**Results:**

This study ultimately included 1,388 patients and their corresponding images. A total of 851 radiomics features were extracted for each binary classification problems. Following feature selection, 18 to 76 features were retained from each groups. The area under the receiver operating characteristic curve (AUC) for the validation cohorts was 0.834 (95% CI: 0.779–0.885) for the LFSI grade 0 vs. LFSI grade 1, 0.771 (95% CI: 0.713–0.835) for LFSI grade 1 vs. LFSI grade 2, and 0.830 (95% CI: 0.762–0.885) for LFSI grade 2 vs. LFSI grade 3.

**Conclusion:**

Machine learning models based on ultrasound radiomics are feasible for classifying different stages of liver fibrosis caused by *Schistosoma* infection.

## Author summary

Schistosomiasis is a devastating disease caused by parasitic worms, leading to stunting, reduced learning ability in children, and impaired work capacity in adults. Currently, there is no ideal staging system to assess schistosomiasis-related liver conditions. Advances in machine learning can help us understand and evaluate liver ultrasound images in entirely new dimensions. Regions with high infection rates are predominantly underdeveloped and characterized by a scarcity of medical resources, where B-mode ultrasound equipment serves as one of the primary diagnostic tools. This study aims to develop an intelligent recognition model based on ultrasound radiomics and machine learning to provide a basis for the ultrasound diagnosis of schistosomiasis.

## Introduction

Schistosomiasis, a neglected tropical disease, is prevalent in 76 regions globally and impacts the lives of 250 million people, highlighting the need for intensified interventions [1,2]. Since 2000, the incidence of schistosomiasis in Southeast Asia has significantly declined, yet the disease remains rampant in certain areas, particularly with *Schistosoma japonicum* and *Schistosoma mekongi*. *Schistosoma japonicum* usually causes more severe diseases than *Schistosoma mekongi*, including liver fibrosis, abdominal pain, diarrhea, liver cirrhosis, and portal hypertension [1]. This study focused on *Schistosoma japonicum*. *Schistosomiasis japonica* has three clinical manifestation types: acute, chronic, and advanced. Acute schistosomiasis is usually caused by many schistosome cercariae infections within a short period [2]. Chronic schistosomiasis results from repeated infections with schistosome cercariae and a lack of timely and thorough treatment. Long-term chronic schistosomiasis infection can lead to liver fibrosis, portal hypertension, and other symptoms, with a chance of progressing to advanced schistosomiasis [3,4]. Research indicates that treatment can reduce *Schistosoma* infection and its related conditions such as hepatomegaly, splenomegaly, and periportal fibrosis, based on disease progression [3–5]. Therefore, effective schistosomiasis management relies on two key factors: early diagnosis of *Schistosoma japonicum* infection to reduce symptoms and halt disease progression and differentiating between its chronic and advanced stages for targeted treatment [4,5]. The liver fibrosis grading in Schistosomiasis Japonica is a valuable indicator for understanding the disease stage in patients [6,7]. It typically involves a series of medical examinations and assessments, including clinical history and physical examination, blood tests, imaging studies, liver tissue biopsy, and elastography [6,7]. B-mode ultrasound (US) examination is a non-invasive test (NIT) that allows for the observation of the liver fibrosis progression in schistosomiasis [6,7]. During schistosomiasis epidemiological surveys, US examinations can rapidly examine a large population and provide immediate results [8]. The correctness of schistosomiasis US diagnosis is related to the experience and proficiency of the US operator, which limits its potential for broader application and promotion [9].

Current international ultrasonography diagnostic standards for schistosomiasis were established at a WHO conference in 1996, primarily based on the infection caused by *Schistosoma mansoni* and *Schistosoma haematobium* [9,10]. The pathological progression of Schistosomiasis japonica differs from that of other schistosomiasis subtypes, especially in terms of liver fibrosis. The utility of ultrasonography diagnosis for schistosomiasis japonica was affirmed by the Chinese US Imaging Diagnosis Experience Exchange Meetings in 1996 and 2003, and the criteria for detection and diagnosis were standardized [11]. The classification of liver fibrosis in Schistosomiasis japonica is different from that of common chronic liver diseases, so the division of ultrasonographic parenchymal echo indicating liver fibrosis due to *Schistosoma* infection (LFSI) into four stages was confirmed by this meeting (Grade 0 to Grade 3) [11]. Although both Schistosomiasis japonica and Schistosomiasis mansoni result in liver fibrosis, they exhibit distinct differences in the focal points of lesions and the evaluation of disease progression through imaging diagnostics. In Schistosomiasis japonica, the primary focus lies in assessing the extent of fibrosis throughout the entire liver. Schistosomiasis mansoni places greater emphasis on specifically evaluating the severity of periportal fibrosis, concentrating on the areas surrounding the portal veins within the liver [9–12].

Converting clinical and imaging data into clinically valuable information remains a significant challenge [13,14]. Artificial intelligence has been increasingly applied in the field of hepatic disease in recent years, offering new perspectives and opportunities for research [13]. Radiomics is a novel approach to enhancing the accuracy of medical imaging diagnosis, which refers to the extraction of high-throughput features from medical images and their analysis and evaluation based on practical problems, ultimately used for disease-assisted diagnosis, classification, or prediction [14]. In 2016, the Image Biomarker Standardization Initiative was released, enhancing the standardization and reproducibility of radiomics methodology [15,16].

The primary objective of this study is to create an auxiliary diagnostic framework using ultrasonography images, an affordable medical diagnostic technology, in conjunction with radiomics and machine learning models.

## Methods

### Data acquisition and exclusion criteria

**Fig 1:**
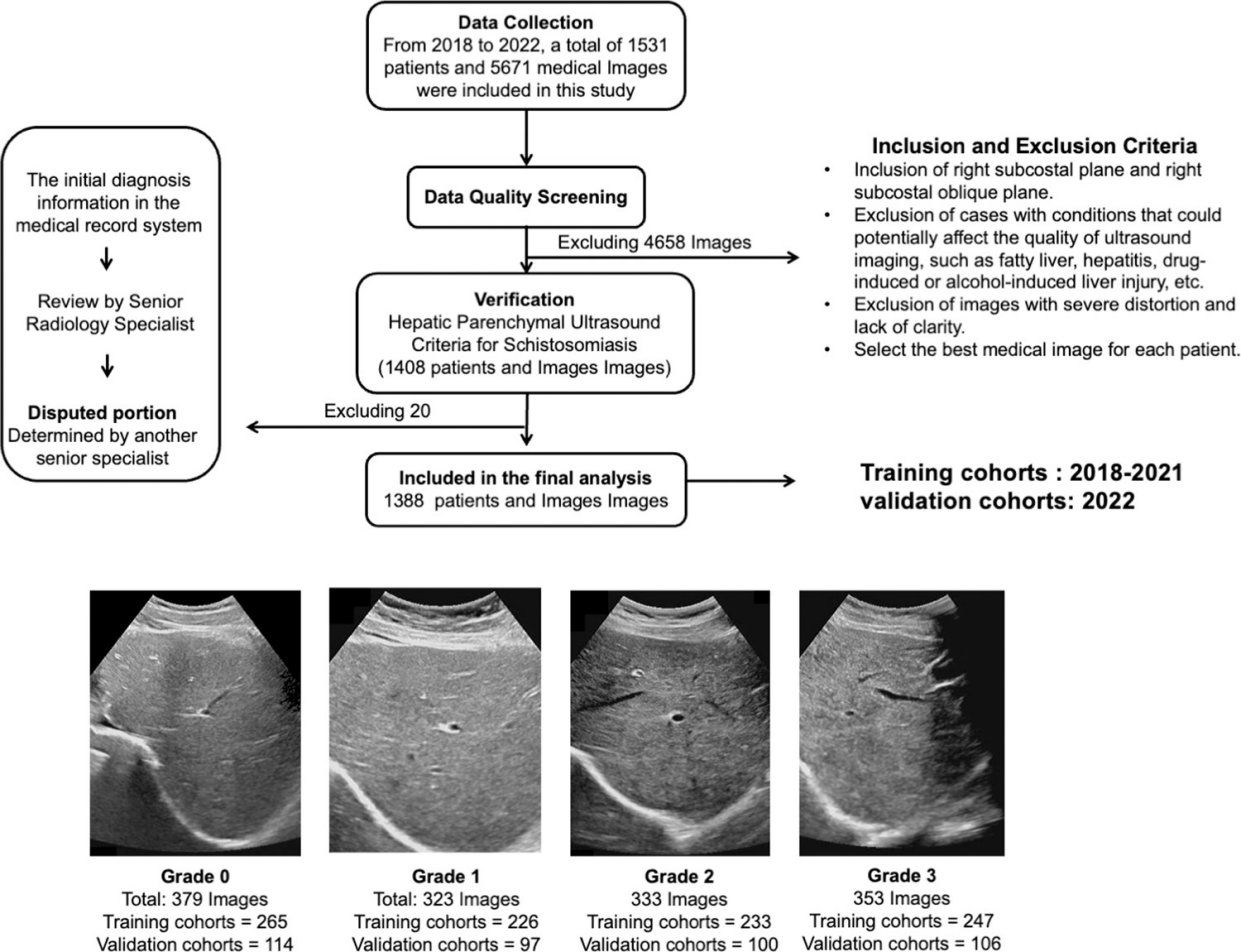
Flow diagram of the recruitment pathway.

A total of 1,531 patients and 5,671 medical images were included in this study from the DuChang City Second People’s Hospital in Jiangxi Province, China, spanning from January 2018 to December 2022. To ensure the accuracy of the data included, this study adopted inclusion and exclusion criteria: (I) Positive serological diagnosis of schistosomiasis, (II) Inclusion of right subcostal plane and right subcostal oblique plane, (III) Exclusion of cases with conditions affecting US imaging quality, like fatty liver, hepatitis, drug-induced or alcohol-induced liver injury, etc. A common reason is that severe fatty liver leads to inaccurate staging of liver fibrosis, and (IV) Exclusion of images with severe distortion and lack of clarity (Fig 1).

To ensure the accuracy of grading liver fibrosis caused by schistosome infection, all data were revalidated after de-identification. First, the initial diagnosis information from the medical record system was recorded. Then, a radiologist with over 10 years of relevant work experience re-evaluated the ultrasound images. Finally, for disputed parts in the initial diagnosis and re-evaluation of schistosome-induced liver fibrosis grading, another senior expert assessed and re-discussed whether to include them (Fig 1). This research was completed with reference to the CheckList for EvaluAtion of Radiomics research (CLEAR) and Radiomics Quality Score (RQS) (S1 File) [17,18].

Data from 2018 to 2021 comprised the training cohort, and data from 2022 formed the validation cohort (Fig 1). The imaging equipment utilized was the Philips EPIQ5, with the Philips C51 curvilinear probe (3.5–5 MHz). Images were exported in DICOM format. LFSI grade 0 represents healthy individuals, with uniform liver parenchyma echo and fine or slightly thicker bright spots. LFSI grade 1 represents the earliest stage of liver damage. Liver parenchymal echo is uneven, and the bright spots become coarser [8]. This type can be seen at various stages of schistosomiasis, and the correct diagnosis requires combining other information. Liver function may still be relatively normal, and patients may not experience significant symptoms. LFSI grade 2 is a moderate stage of liver damage. Liver function may begin to decline, and patients may experience symptoms such as fatigue, abdominal discomfort, and hepatomegaly [8]. The liver parenchymal echo is unevenly distributed, with enhanced bright spots, forming a fish scale-shaped pattern. A fine mesh-like echo can be seen throughout the entire liver. LFSI grade 3 usually represents advanced schistosomiasis liver damage (Fig 1) [8,12]. The liver parenchymal echo displays an uneven pattern, with thicker, more intense bright spots and higher-pitched echoes. Echoes form a densely packed, coarse network-like high echo band [11].

The ultrasonography diagnosis of LFSI was categorized into four stages (Grade 0 vs. Grade 3). Grade 0 is health person. In this research, the data was divided into six groups: Group 1 (Grade 0 vs. Grade 1), Group 2 (Grade 0 vs. Grade 2), Group 3 (Grade 0 vs. Grade 3), Group 4 (Grade 1 vs. Grade 2), Group 5 (Grade 1 vs. Grade 3), and Group 6 (Grade 2 vs. Grade 3). The gender distribution and average age between the training and validation cohorts did not exhibit significant differences. The distribution of LFSI grades in the training and validation cohorts is presented in S2 Data.

### Image processing and segmentation

Each region of interest (ROI) was delineated by two researchers using ITK-SNAP Version 3.8.0 in 2022[19]. Both researchers had received specialized training in data annotation and US imaging. The images were reconstructed into grayscale images using the weighted average method, and each voxel size was adjusted to 1 mm × 1 mm × 1 mm using linear interpolation. Intraclass Correlation Coefficients (ICCs) were utilized to assess annotation agreement analysis, determining the efficacy and reliability of the annotation scheme. Features with an ICC greater than 0.8 were incorporated into the subsequent phase [20].

### Feature extraction and selection

The computational platform is outfitted with a 16-core AMD EPYC 7742 processor and 32 GB of RAM. The open-source Pyradiomics 2.1.2 toolkit (https://github.com/AIM-Harvard/pyradiomics) in Python 3.7 (www.python.org) was used to extract radiomic features from 2 groups of ROIs, including first-order statistical features, texture features, and wavelet features [21]. Radiomics features are typically named following a structured convention that provides information about their characteristics. The naming format includes the feature class (e.g., first order, Gray Level Co-occurrence Matrix, and Gray Level Run Length Matrix), the specific feature name (e.g., mean, entropy and contrast), and any additional parameters or preprocessing steps used in their calculation. Feature selection was performed only on the training cohorts, including the Mann-Whitney U test and LASSO [22].

### Machine learning model construction and interpretability

Based on the data obtained in the feature selection phase, this research uses the scikit-learn toolkit for machine learning modeling, including Support Vector Machine (SVM), Random Forest (RF), Stochastic Gradient Descent (SDG), K-Nearest Neighbors (KNN), eXtreme Gradient Boosting (XGBoost), and Light Gradient Boosting Machine (LR) [23]. To identify the optimal machine learning model, this study uses AUC as the primary evaluation metric, supplemented by Precision, F1-Score, Accuracy, Sensitivity, and Specificity as additional evaluation criteria [24]. To ensure the best performance of the model, five-fold cross-validation was used to identify the optimal parameters [25], and the Synthetic Minority Oversampling Technique (SMOTE) was employed to address data imbalance issues [26]. In the field of medicine, enhancing the interpretability of models can improve their reliability. Therefore, we employ Shapley Additive exPlanations (SHAP), which can determine the impact of each feature on the model’s predictions [27–29].

### Ethics statement

The Ethical Review Committee of the National Institute of Parasitic Diseases, Chinese Center for Disease Control and Prevention has reviewed this project (Ethics Review Number: 2021019). Prior to participation, all subjects involved in the study were thoroughly informed about the research aims, procedures, potential benefits, and risks. Written informed consent was obtained from each participant, ensuring voluntary participation with a full understanding of their role. No child participants were involved in this study.

## Results

**Fig 2:**
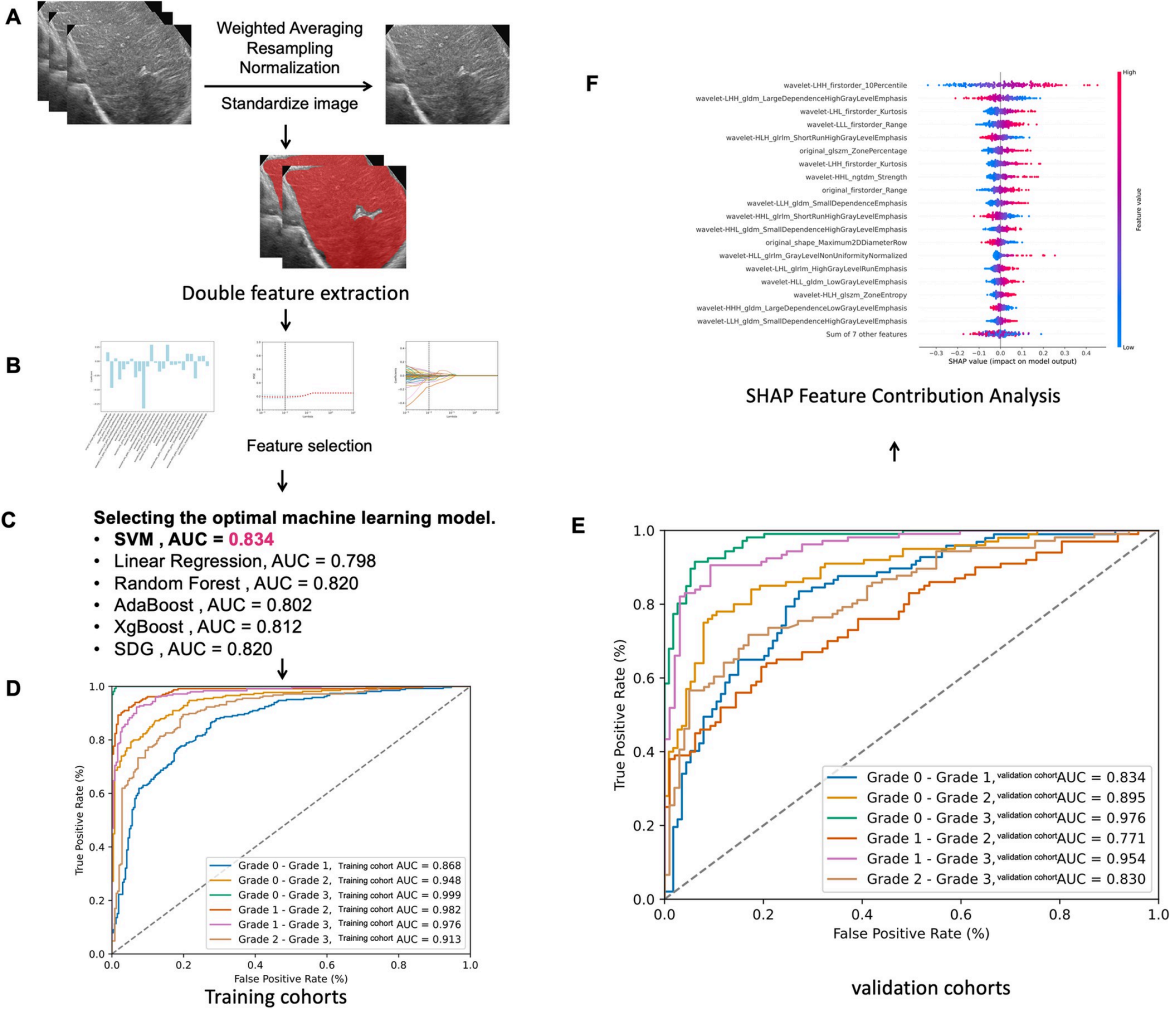
Workflow of radiomics model building and analysis. (A) The US images undergo double annotations by two distinct physicians, and intraclass correlation coefficient (ICC) is 0.956. (B) Feature selection and Rad Score. (C) For the grade 0 vs. grade 1 group, six machine learning models were employed, with SVM emerging as the most superior choice. (D) ROC curves and AUC values for training cohorts (SVM). (E) ROC curves and AUC values for validation cohorts (SVM). (F) Using SHAP to Explain the Contribution of Features to Machine Learning Models.

This study ultimately included 1,388 patients and images. A total of 851 features were extracted from each group of data (Grade 0 vs. Grade 3). After feature selection using the Mann–Whitney U test and LASSO(**Fig 2**), Group 1 (Grade 0 vs. Grade 1) ended up with 26 features, Group 2 (Grade 0 vs. Grade 2) had 31 features, Group 3 (Grade 0 vs. Grade 3) retained 76 features, Group 4 (Grade 1 vs. Grade 2) kept 37 features, Group 5 (Grade 1 vs. Grade 3) had 49 features, and Group 6 (Grade 2 vs. Grade 3) had 18 features. The list of features used for the six data sets is in **S1 Data**.

In the model selection phase, six models were included: SVM, RF, SDG, KNN, XGBoost, and LR. The primary evaluation metric used was the AUC. SVM demonstrates the best performance among different machine learning models (AUC = 0.834) (Table 1).

**Table 1:** Performance of different machine learning models on validation cohorts.

	AUC	Specificity	Sensitivity	Kappa	F1-Score	MCC
SVM	0.834	0.763	0.711	0.475	0.715	0.475
RF	0.821	0.740	0.712	0.472	0.710	0.481
SDG	0.820	0.752	0.700	0.481	0.695	0.472
LR	0.798	0.713	0.693	0.467	0.681	0.468
XGBoost	0.812	0.762	0.695	0.501	0.693	0.475
AdaBoost	0.802	0.731	0.705	0.473	0.723	0.465

Abbreviations: AUC = Area Under the Curve, AUC 95% CI = AUC 95% Confidence Interval, MCC = Matthews Correlation Coefficient, AUPRC = Area Under the Precision-Recall Curve

Table 2 presents six models, each utilizing Support Vector Machines (SVM) as the core classification algorithm. The AUC performance on the validation cohorts of six data groups ranges from 0.771 to 0.976 (Table 2). The AUC for the training set of Group 1 (Grade 0 vs. Grade 1) was 0.868 (95% CI: 0.837, 0.897), and the AUC for the validation set was 0.834 (95% CI: 0.779–0.885), indicating that the model is stable and not overfitted. The narrow confidence intervals suggest low uncertainty in the results. The AUC for the training set of Group 2 (Grade 0 vs. Grade 2) was 0.948 (95% CI: 0.929, 0.964), and the AUC for the validation set was 0.895 (95% CI: 0.845, 0.934). Although the validation set AUC is slightly lower than the training set, it still demonstrates that the model has good generalization ability. The AUC for the training set of Group 3 (Grade 0 vs. Grade 3) was 0.999 (95% CI: 0.999, 0.999), indicating that the model has extremely high accuracy. The AUC for the training set of Group 4 (Grade 1 vs. Grade 2) was 0.982 (95% CI: 0.970, 0.992), while the AUC for the validation set was 0.771 (95% CI: 0.713, 0.835). Model 5 (Grade 1 vs. Grade 3) performed well on the training set, with an AUC of 0.976 (95% CI: 0.963, 0.986), and an AUC of 0.954 (95% CI: 0.927, 0.978) on the validation set. Model 6 (Grade 2 vs. Grade 3) performed excellently on the training set, with an AUC of 0.913 (95% CI: 0.886, 0.937), demonstrating good performance, and the AUC on the validation set was 0.830 (95% CI: 0.762, 0.885).

**Table 2:** Performance of SVM in 6 sets of model training and validation cohorts.

Groups	Dataset Split	AUC	AUC 95% CI	Sensitivity	Specificity	Accuracy	Precision	F1-Score	Kappa
Grade 0 vs. Grade 1	Training	0.868	[0.837, 0.897]	0.792	0.792	0.792	0.792	0.792	0.585
	validation	0.834	[0.779, 0.885]	0.711	0.763	0.739	0.719	0.715	0.475
Grade 0 vs. Grade 2	Training	0.948	[0.929, 0.964]	0.860	0.883	0.872	0.880	0.870	0.743
	validation	0.895	[0.845, 0.934]	0.840	0.807	0.822	0.792	0.816	0.645
Grade 0 vs. Grade 3	Training	0.999	[0.999, 0.999]	0.996	0.989	0.992	0.989	0.992	0.985
	validation	0.976	[0.959, 0.989]	0.925	0.895	0.909	0.891	0.907	0.818
Grade 1 vs. Grade 2	Training	0.982	[0.970, 0.992]	0.914	0.953	0.933	0.951	0.932	0.867
	validation	0.771	[0.713, 0.835]	0.660	0.722	0.690	0.710	0.684	0.381
Grade 1 vs. Grade 3	Training	0.976	[0.963, 0.986]	0.899	0.935	0.917	0.933	0.915	0.834
	validation	0.954	[0.927, 0.978]	0.906	0.835	0.872	0.857	0.881	0.743
Grade 2 vs. Grade 3	Training	0.913	[0.886, 0.937]	0.834	0.826	0.830	0.827	0.831	0.660
	validation	0.830	[0.762, 0.885]	0.736	0.750	0.743	0.757	0.746	0.485

**Fig 3:**
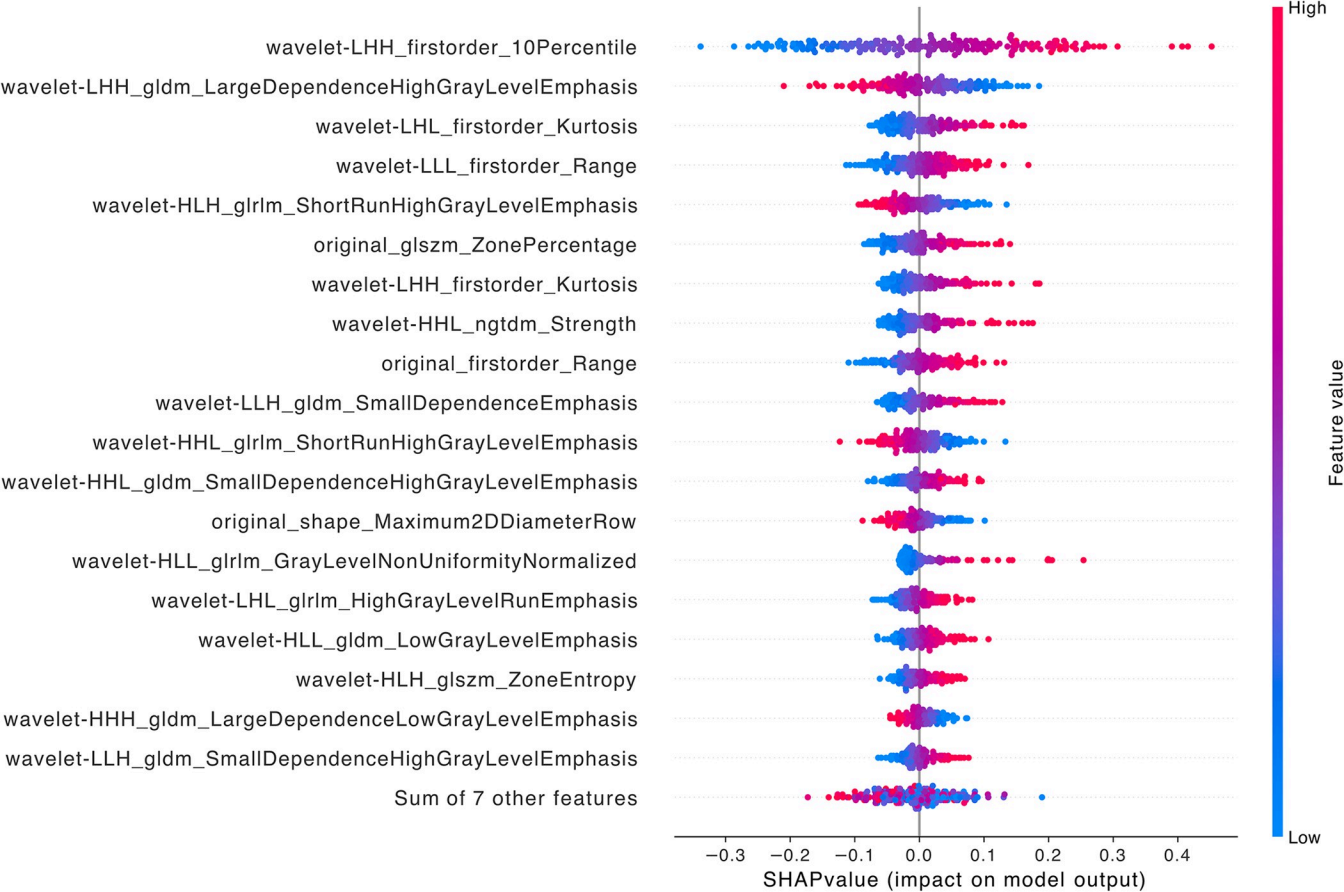
SHAP summary diagram of radiomic features of LFSI grade 0 vs. LFSI grade 1. Each dot represents a patient, with the color gradient from blue (low) to red (high) indicating the feature value. The x-axis represents SHAP values, which indicate the degree of contribution to the model. The y-axis represents feature names, displaying the top 20 contributing feature values.

When comparing LFSI grade 0 with grade 1, the top five features are wavelet-LHH_firstorder_10Percentile, *wavelet-LHH_gldm_LargeDependenceHighGrayLevelEmphasis*, *wavelet-LHL_firstorder_Kurtosis*, *wavelet-LLL_firstorder_Range*, and *wavelet-HLH_glrlm_ShortRunHighGrayLevelEmphasis* (Fig 3). All of these are wavelet transform features, including 3 first-order features, 1 gray level dependence matrix feature (GLDM), and 1 gray level run length matrix feature (GLRLM). First Order Features are a set of features directly extracted from the statistical distribution of image pixel gray values, without involving spatial relationships between pixels. They can describe the basic properties of an image, such as brightness and contrast, from an overall perspective and are often used to complement other features to improve descriptive power. The calculation process of GLDM considers the magnitude relationship between pixel values, not just the number of clusters. This makes GLDM better at reflecting texture details compared to other statistics-based texture features (such as gray level co-occurrence matrix). GLRLM is a texture analysis technique based on run-length encoding, which can effectively characterize the statistical patterns of gray value and pixel run-length distribution in an image, and has good performance in representing complex texture patterns such as roughness and stripes [30–32].

**Fig 4:**
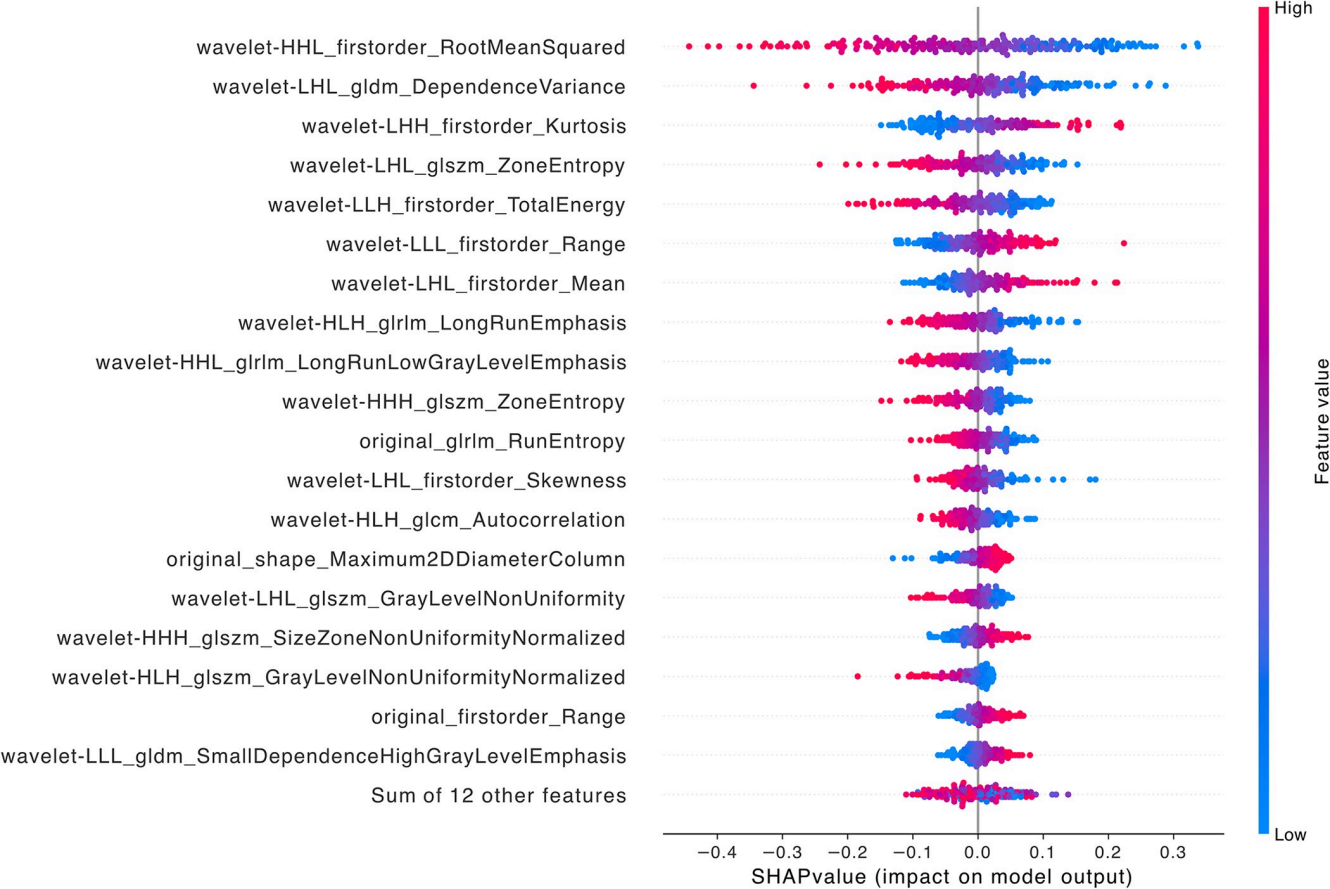
SHAP summary diagram of radiomic features of LFSI grade 0 vs. LFSI grade 2. Each dot represents a patient, with the color gradient from blue (low) to red (high) indicating the feature value. The x-axis represents SHAP values, which indicate the degree of contribution to the model. The y-axis represents feature names, displaying the top 20 contributing feature values.

When comparing LFSI grade 0 with grade 2, the features with the highest contribution are *wavelet-HHL_firstorder_RootMeanSquared*, *wavelet-LHL_gldm_DependenceVariance*, *wavelet-LHH_firstorder_Kurtosis*, *wavelet-LHL_glszm_ZoneEntropy*, and *wavelet-LLH_firstorder_TotalEnergy* (Fig 4). All five radiomics features are wavelet transform features, including three first-order features, one gray-level dependence matrix (GLDM) feature, and one gray-level size zone matrix (GLSZM) feature [21]. GLSZM is an important method for describing the distribution characteristics of gray value regions in images and can effectively characterize complex texture patterns such as speckled and lumpy patterns [21,30–32].

**Fig 5:**
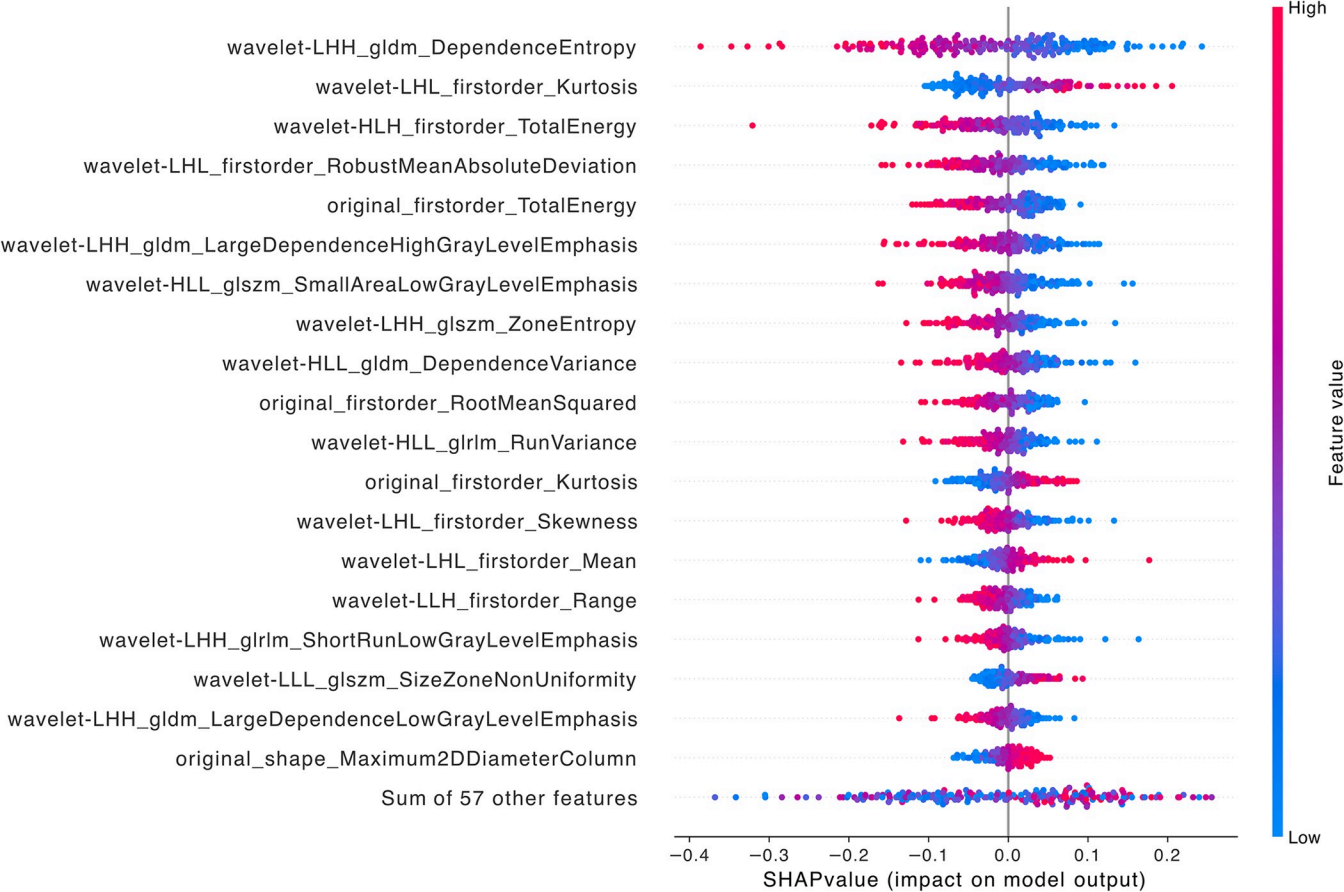
SHAP summary diagram of radiomic features of LFSI grade 0 vs. LFSI grade 3. Each dot represents a patient, with the color gradient from blue (low) to red (high) indicating the feature value. The x-axis represents SHAP values, which indicate the degree of contribution to the model. The y-axis represents feature names, displaying the top 20 contributing feature values.

When comparing LFSI grade 0 with grade 3, the top five features contributing to the model are *wavelet-LHH_glam_DependenceEntropy*, *wavelet-LHL_firstorder_Kurtosis*, *wavelet-HLH_firstorder_TotalEnergy*, *wavelet-LHL_firstorder_RobustMeanAbsoluteDeviation*, and *original_firstorder_TotalEnergy*. Among these 5 features, there is 1 original image feature and 4 wavelet transform features. The wavelet transform features include 3 first-order features and 1 gray level dependence matrix feature [21,30–32]. Group 3 (Grade 0—Grade 3) had slightly higher SHAP values for each feature, resulting in the highest AUC value for the final model (Fig 5).

**Fig 6:**
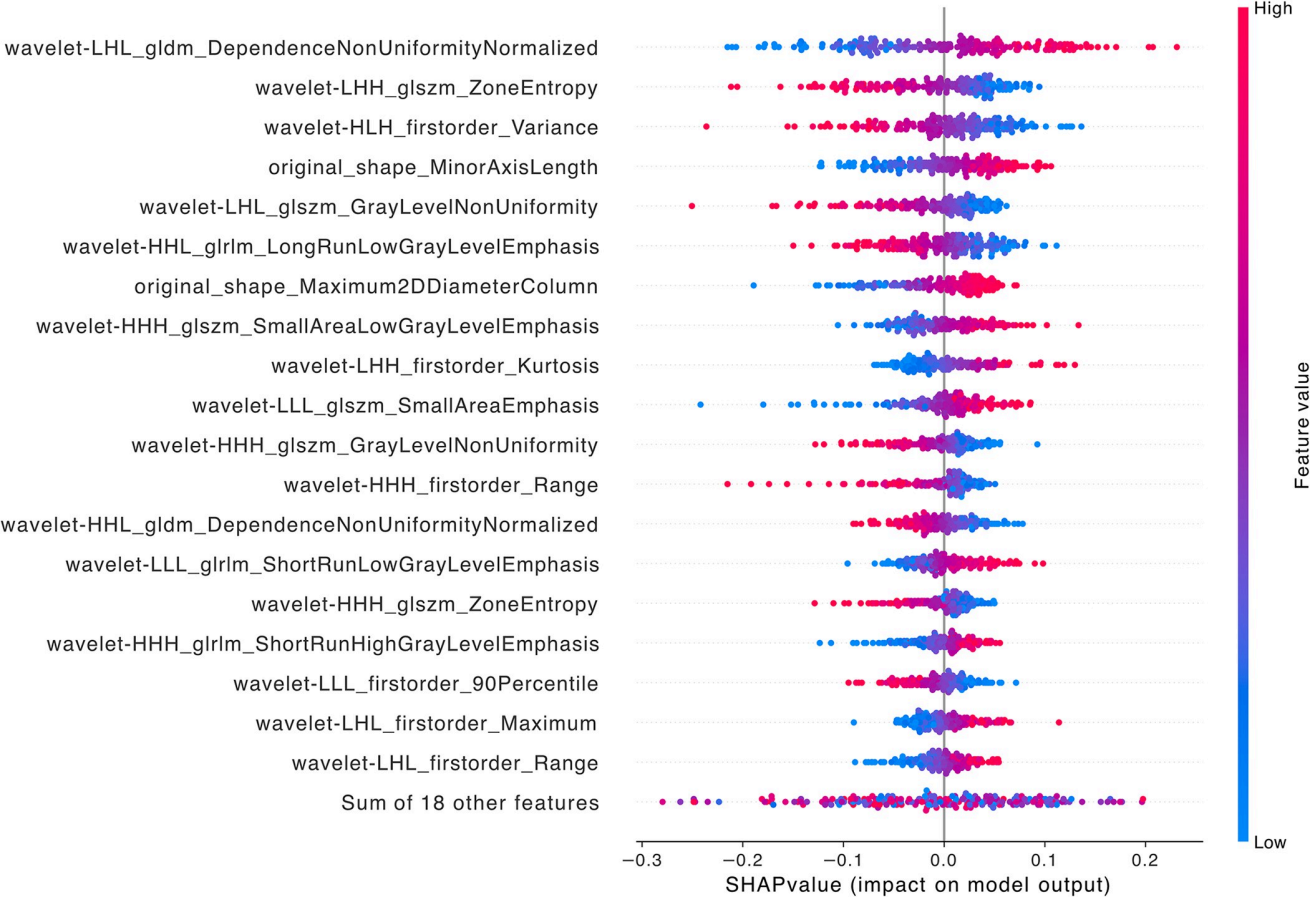
SHAP summary diagram of radiomic features of LFSI grade1 vs. LFSI grade 2. Each dot represents a patient, with the color gradient from blue (low) to red (high) indicating the feature value. The x-axis represents SHAP values, which indicate the degree of contribution to the model. The y-axis represents feature names, displaying the top 20 contributing feature values.

When comparing LFSI grade 1 with grade 2, the top five features with the highest contribution are *wavelet-LHL_gldm_DependenceNonUniformityNormalized*, *wavelet-LHH_gIszm_ZoneEntropy*, *wavelet-HLH_firstorder_Variance*, *original_shape_MinorAxisLength*, and *wavelet-LHL_gIszm_GrayLevelNonUniformity* (Fig 6). Among these 5 features, there is 1 original image feature and 4 wavelet transform features. The wavelet transform features include 1 first-order feature, 1 GLDM feature, and 2 GLSZM features [21,30–32].

**Fig 7:**
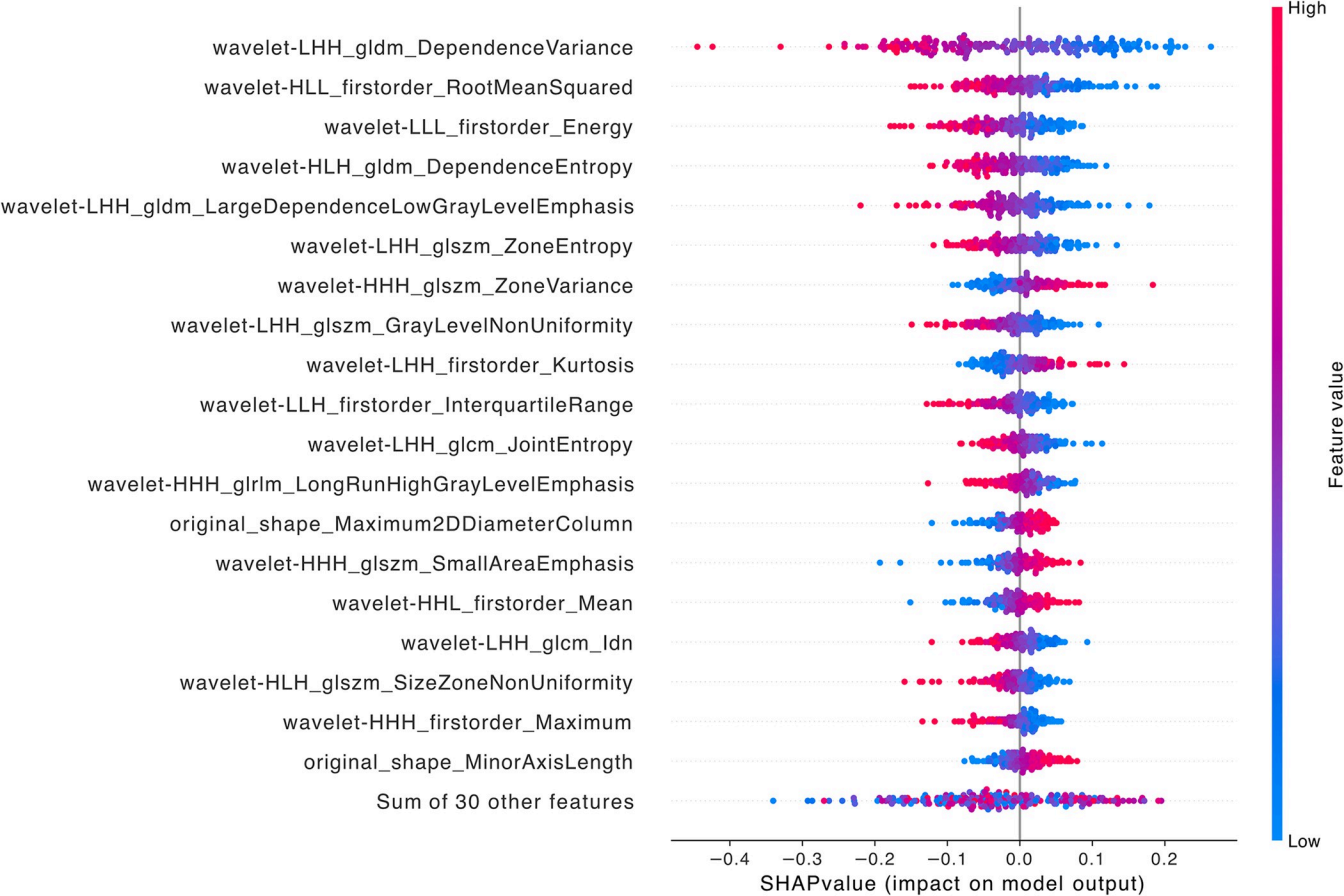
SHAP summary diagram of radiomic features of LFSI grade 1 vs. LFSI grade 3. Each dot represents a patient, with the color gradient from blue (low) to red (high) indicating the feature value. The x-axis represents SHAP values, which indicate the degree of contribution to the model. The y-axis represents feature names, displaying the top 20 contributing feature values.

When comparing LFSI grade 1 with grade 3, the features with the highest contribution are *wavelet-LHH_glam_DependenceVariance*, *wavelet-HLL_firstorder_RootMeanSquared*, *wavelet-LLL_firstorder_Energy*, *wavelet-HLH_gldm_DependenceEntropy*, and *wavelet-LHH_gldm_LargeDependenceLowGrayLevelEmphasis* (Fig 7). All 5 features are wavelet transform features, including 2 first-order features and 3 GLDM features [21,30–32].

**Fig 8:**
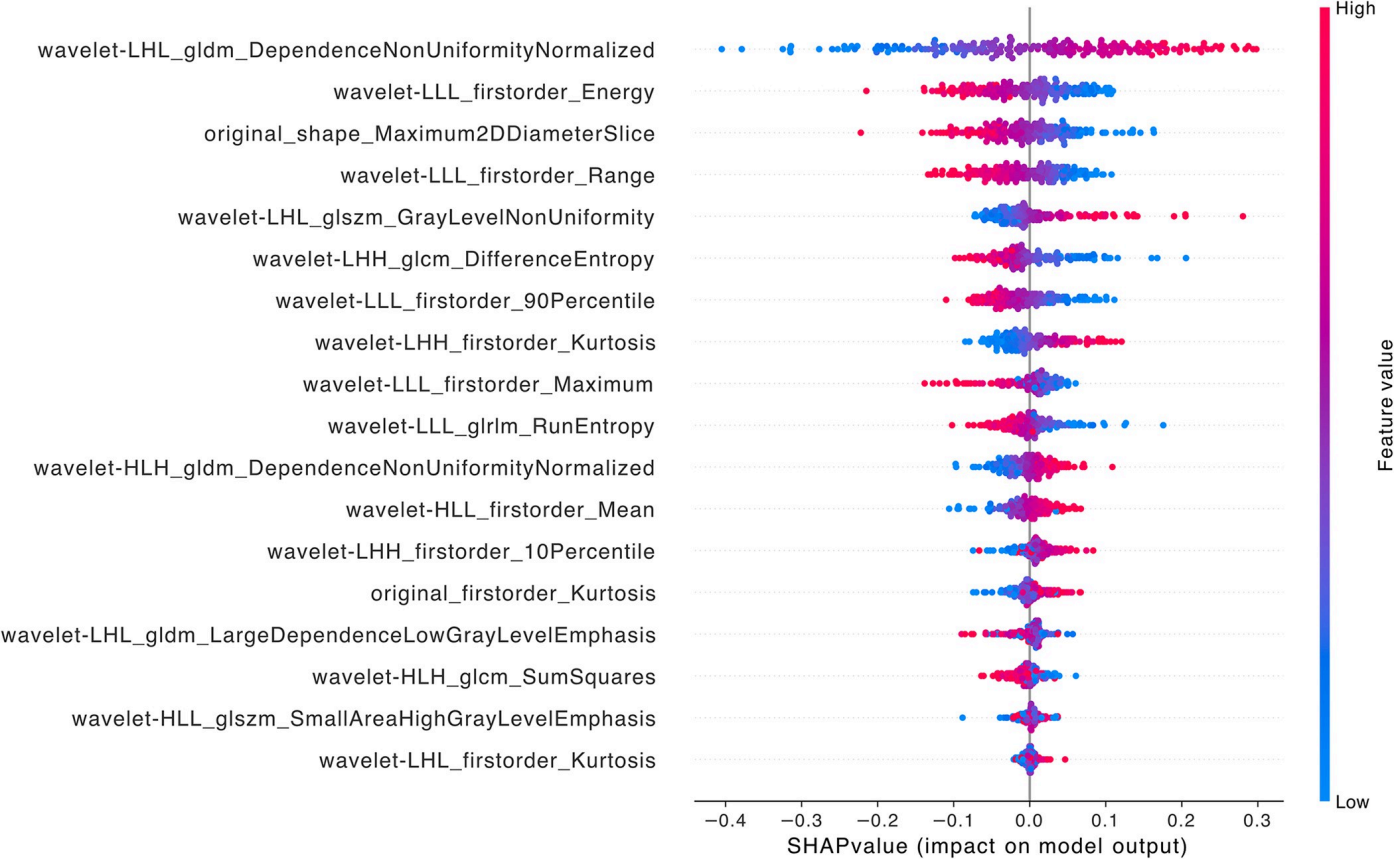
SHAP summary diagram of radiomic features of LFSI grade 2 vs. LFSI grade 3. Each dot represents a patient, with the color gradient from blue (low) to red (high) indicating the feature value. The x-axis represents SHAP values, which indicate the degree of contribution to the model. The y-axis represents feature names, displaying the top 20 contributing feature values.

When comparing LFSI grade 3 with grade 4, the top five features ranked by contribution are *wavelet-LHL_gldm_DependenceNonUniformityNormalized*, *wavelet-LLL_firstorder_Energy*, *original_shape_Maximum2DDiameterSlice*, *wavelet-LLL_firstorder_Range*, and *wavelet-LHL_glszm_GrayLevelNonUniformity* (Fig 8). Among these 5 features, there is 1 original image feature and 4 wavelet transform features. The 4 wavelet transform features include 2 first-order features, 1 GLDM feature, and 1 GLSZM feature [21,30–32].

*Wavelet-LHH_firstorder_Kurtosis* is present in 5 groups, which may indicate the wide applicability of this Radiomics feature in schistosomiasis hepatic fibrosis. We observed that *wavelet-LHH_firstorder_10Percentile*, *wavelet-HHL_firstorder_RootMeanSquared*, *wavelet-LHH_gldm_DependenceEntropy*, *wavelet-LHL_gldm_DependenceNonUniformityNormalized*, and *wavelet-LHH_gldm_DependenceVariance* had the highest importance. *Wavelet-LHH_firstorder_10Percentile* and *wavelet-HHL_firstorder_RootMeanSquared* is a first-order statistical feature extracted using wavelet filters [30–32]. *Wavelet-LHH_firstorder_10Percentile* is a texture feature calculated from the wavelet-transformed image, representing the value below which 10 percent of the pixel values in the low-high-high frequency band fall. *Wavelet-HHL_firstorder_RootMeanSquared* is a texture feature that calculates the root mean squared value of pixel intensities in the high-high-low frequency band of a wavelet-transformed image, indicating the square root of the average squared intensity values. *Wavelet-LHH_gldm_DependenceEntropy*, *wavelet-LHL_gldm_DependenceNonUniformityNormalized*, and *wavelet-LHH_gldm_DependenceVariance* were calculated based on the Gray Level Dependence Matrix (GLDM). These metrics evaluate the texture and structural features within an image by quantifying the dependencies among different gray levels within the image [21]. *Wavelet-LHH_gldm_DependenceEntropy* is a measure of the randomness or unpredictability of pixel dependencies within the low-high-high frequency band of a wavelet-transformed image. *Wavelet-LHL_gldm_DependenceNonUniformityNormalized* measures the normalized value of dependency non-uniformity, reflecting whether the distribution of texture dependencies in an image is uniform. *Wavelet-LHH_gldm_DependenceVariance* measures the degree of variation in the dependency relationships among elements in image textures. Put simply, it looks at how much the relationship between each small block (or pixel) and the surrounding blocks varies within an image [30].

**Fig 9:**
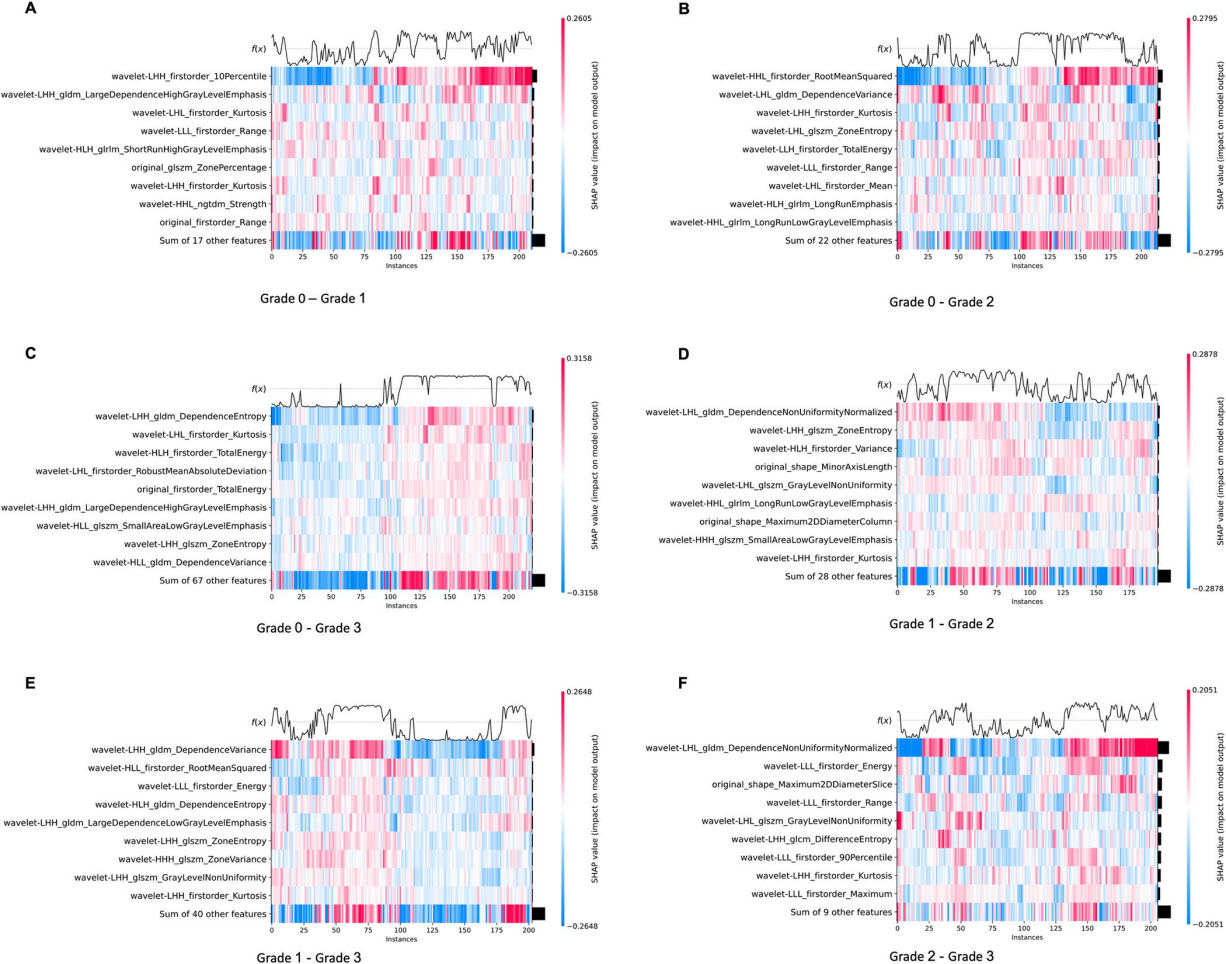
SHAP Heatmap of Radiomics feature contribution for six groups. Fig 9A–9F represented the differences in feature contributions for each sample in the form of heatmaps. (A) Grade 0 vs. Grade 1, (B) Grade 0 vs. Grade 2, (C) Grade 0 vs. Grade 3, (D) Grade 1 vs. Grade 2, (E) Grade 1 vs. Grade 3, and (F) Grade 2 vs. Grade 3. Each row represents a sample, and each column represents a feature. The color coding in the heatmap indicates the contribution of each feature to the model’s output. Typically, positive values (in red) signify a positive contribution to the prediction, while negative values (in blue) indicate a negative contribution. The intensity of the color represents the degree of contribution.

The heatmaps help understand the model’s decision-making process and assist in identifying the importance of input features. By using SHAP heatmaps, it is possible to quickly locate samples with abnormal states. The SHAP value distributions for Grade 0 vs. Grade 3 and Grade 1 vs. Grade 3 are uniform, resulting in higher AUC values. The other four SVM groups have relatively more outliers (Fig 9).

## Discussion

Schistosomiasis japonica is a parasitic disease that poses a significant public health challenge across several Asian countries, including Japan, China, the Philippines, and Indonesia [1–3]. According to the 2022 National Progress Report on Schistosomiasis Control in China, there are a total of 28,565 cases of advanced schistosomiasis nationwide [33]. While schistosomiasis is at a low prevalence level across the country, there has been a resurgence in certain local areas. It is essential to enhance schistosomiasis detection and treatment capabilities, particularly for advanced patients. On-site investigations showed that ultrasound, a widely available medical infrastructure in areas where schistosomiasis is prevalent, enables convenient and non-invasive diagnostic procedures. Developing US imaging-based diagnostic support tools is a solution that meets local needs. Research has indicated that after treatment with Praziquantel, patients with schistosomiasis experienced positive improvements in the degree of liver fibrosis [34]. These improvements were more apparent in grade 1 vs. grade 2 [34]. Improving the accuracy of the clinical decision-making process will help enhance patients’ treatment plans and reduce their disease burden.

**Fig 10:**
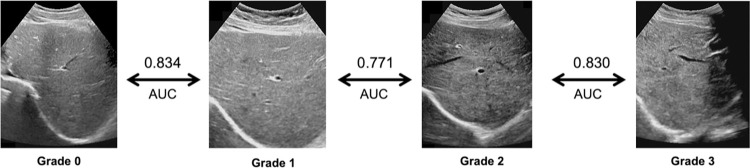
Radiomics-based SVM machine learning model for distinguishing key stages of liver fibrosis caused by *Schistosoma* infection.

This project selected 1,388 images from a total of 5,671 US images. Among them, there were 379 LFSI grade 0 images, 323 LFSI grade 1 images, 333 LFSI grade 2 images, and 353 LFSI grade 3 images. After ROI labeling, feature extraction, and feature selection stages, a total of 18 to 76 features were extracted in the end. The AUC values for all groups were above 0.771, indicating the model’s strong predictive ability. The AUC of the validation cohorts for the healthy group vs. LFSI grade 1 is 0.834 (95% CI: 0.779–0.885). The AUC of the validation cohorts for LFSI grade 1 vs. LFSI grade 2 is 0.771 (95% CI: 0.713–0.835). The AUC of the validation cohorts for LFSI grade 2 vs. LFSI grade 3 is 0.830 (95% CI: 0.762–0.885) (Fig 10). These three classification models are the focus of this study. The good and stable AUC indicates that the models could distinguish different stages of LFSI (Fig 10). Group 4 (Grade 1 vs. Grade 2) has the lowest AUC (0.771), and this classification problem is also the most challenging in clinical differential diagnosis. Group 1 (Grade 0 vs. Grade 1), Group 2 (Grade 0 vs. Grade 2), and Group 3 (Grade 0 vs. Grade 3) have AUC values exceeding 0.834, suggesting that the model can distinguish between healthy individuals and schistosomiasis patients with high accuracy. Among these three models, Group 1 had the lowest AUC, while Group 3 had the highest AUC, which is consistent with the experience of clinical radiologists.

IBSI and Pyradiomics are tools developed to enhance the interpretability of radiomic features in machine learning, where each extracted feature has its mathematical significance. We observed that *wavelet-LHH_firstorder_10Percentile*, *wavelet-HHL_firstorder_RootMeanSquared*, *wavelet-LHH_gldm_DependenceEntropy*, *wavelet-LHL_gldm_DependenceNonUniformityNormalized*, and *wavelet-LHH_gldm_DependenceVariance* had the highest importance. *Wavelet-LHH_gldm_DependenceEntropy*, *wavelet-LHH_gldm_DependenceVariance*, and *wavelet-LHL_gldm_DependenceNonUniformityNormalized* may be associated with the formation of densely packed, coarse network-like high echo bands in the liver parenchymal echo. *Wavelet-HHL_firstorder_RootMeanSquared* and *wavelet-LHL_gldm_DependenceNonUniformityNormalized* may be associated with the liver parenchymal echo being unevenly distributed, and a fine mesh-like echo can be seen throughout the entire liver. *Original_shape_MinorAxisLength*, *original_shape_Maximum2DDiameterColumn*, and *original_shape_Maximum2DDiameterSlice* are all shape features used to describe the shape and size of the Region of Interest (ROI). They may have potential associations with hepatomegaly (enlarged liver).

There are several serological models for diagnosing liver fibrosis that have been established, including the FibroTest, HepaScore [35], FIBROS Spect II, AST to platelet ratio index (APRI) [36], and fibrosis index based on the four factors (FIB-4) [37,38] etc. Thierry Poynard’s research indicates that for diagnosing advanced liver fibrosis (METAVIR scoring system F0–F1 vs. F2–F4), the FibroTest had an AUC of 0.84 (CI 0.79–0.86), while liver stiffness measurement (LSM) had an AUC of 0.89 (CI 0.83–0.96) [35]. In contrast to FibroTest and LSM, this study possesses the capability to provide a more precise evaluation of the stages of liver fibrosis in schistosomiasis. Each of these options has its pros and cons. Considering the uncertainties in the clinical diagnosis and treatment environment, combining serological, imaging, and interview-based approaches can provide a more comprehensive assessment of the patient’s condition [39].

The ultimate objective of this study is its application in field diagnostics, hence there is much room for improvement in the future. Firstly, multicenter prospective data is crucial to improving model quality, enhancing the model’s generalizability [40]. Secondly, this study used a relatively loose definition of liver fibrosis. Future research could investigate the imaging characteristics of liver fibrosis using standardized ultrasound scanning protocols. Thirdly, developing automated segmentation algorithms with models such as U-net is essential for implementing automated diagnostic systems on-site [41]. Fourthly, studies have indicated that integrating imaging characteristics of the spleen and liver can enhance the performance of machine learning models [42]. Fifthly, data-driven radiomics methods are inherently challenging to interpret in the context of their underlying biological and immunological mechanisms, which constrains the explainability and broader clinical adoption of radiomics models. In the future, there are many possibilities for exploration in the radiomics practice of schistosomiasis-related liver diseases [40,43–49].

## Conclusion

The primary contribution of this research is the differentiation of the four stages of liver fibrosis caused by *Schistosoma* infection using quantitative ultrasound image texture features and machine learning models. The radiomics features and machine learning models developed in this study have significant potential to assist in clinical decision-making. They not only have the potential to distinguish between healthy individuals and patients with liver fibrosis due to *Schistosoma* infection (LFSI) but also differentiate between mild LFSI and severe LFSI.

## Supporting information

S1 File ICMJE DISCLOSURE FORM, CLEAR checklist, RQS Checklist, and Statement of Informed Consent. (PDF)

S1 Data Features used in SVM modeling. (XLSX)

S2 Data Performance in training and validation cohorts. (XLSX)

S3 Data Feature Date. (XLSX)
