# Supplementary material for: Feasibility of ultrasound radiomics based models for classification of liver fibrosis due to Schistosoma japonicum infection
Source: PLoS Negl Trop Dis. 2024 Jun 13;18(6):e0012235. doi: 10.1371/journal.pntd.0012235 (PMC11207143; doi:10.1371/journal.pntd.0012235)
Supplement: S1 File — (PDF) [file pntd.0012235.s001.pdf]

Supplemental Material 1

1. ICMJE DISCLOSURE FORM
2. CLEAR checklist
3. Radiomics Quality Score(RQS) Checklist
4. Statement of Informed Consent

ICMJE DISCLOSURE FORM

ICMJE DISCLOSURE FORM

Date: 11/1/2023

Your Name: Zhaoyu Guo

Manuscript Title: Novel Radiomics-based model for Diagnosing Schistosomiasis Japonica Hepatic Fibrosis

Manuscript Number (if known): Click or tap here to enter text.

In the interest of transparency, we ask you to disclose all relationships/activities/interests listed below that are related to the content of your manuscript. "Related" means any relation with for-profit or not-for-profit third parties whose interests may be affected by the content of the manuscript. Disclosure represents a commitment to transparency and does not necessarily indicate a bias. If you are in doubt about whether to list a relationship/activity/interest, it is preferable that you do so.

The author's relationships/activities/interests should be defined broadly. For example, if your manuscript pertains to the epidemiology of hypertension, you should declare all relationships with manufacturers of antihypertensive medication, even if that medication is not mentioned in the manuscript.

In item #1 below, report all support for the work reported in this manuscript without time limit. For all other items, the time frame for disclosure is the past 36 months.

		**Name all entities with whom you have this relationship or indicate none (add rows as needed)**	**Specifications/Comments (e.g., if payments were made to you or to your institution)**
**Time frame: Since the initial planning of the work**			
1	All support for the present manuscript (e.g., funding, provision of study materials, medical writing, article processing charges, etc.) **No time limit for this item.**	☒ None	
			Click the tab key to add additional rows.
**Time frame: past 36 months**			
**2**	Grants or contracts from any entity (if not indicated in item #1 above).	☒ **None**	
**3**	Royalties or licenses	☒ **None**	

		Name all entities with whom you have this relationship or indicate none (add rows as needed)	Specifications/Comments (e.g., if payments were made to you or to your institution)
**4**	Consulting fees	☒ **None**	
**5**	Payment or honoraria for lectures, presentations, speakers bureaus, manuscript writing or educational events	☒ **None**	
**6**	Payment for expert testimony	☒ **None**	
**7**	Support for attending meetings and/or travel	☒ **None**	
**8**	Patents planned, issued or pending	☒ **None**	
**9**	Participation on a Data Safety Monitoring Board or Advisory Board	☒ **None**	
**10**	Leadership or fiduciary role in other board, society, committee or advocacy group, paid or unpaid	☒ **None**	

		Name all entities with whom you have this relationship or indicate none (add rows as needed)	Specifications/Comments (e.g., if payments were made to you or to your institution)
**11**	Stock or stock options	☒ **None**	
**12**	Receipt of equipment, materials, drugs, medical writing, gifts or other services	☒ **None**	
**13**	Other financial or non-financial interests	☒ **None**	

Please place an "X" next to the following statement to indicate your agreement:

☒ I certify that I have answered every question and have not altered the wording of any of the questions on this form.

CLEAR checklist

Electronic Supplementary Material S2: CLEAR checklist without explanations

Section	No.	Item	Yes	No	n/a	Page
**Title**						
	1	Relevant title, specifying the radiomic methodology	☑	☐	☐	
**Abstract**						
	2	Structured summary with relevant information	☑	☐	☐	
**Keywords**						
	3	Relevant keywords for radiomics	☑	☐	☐	
**Introduction**						
	4	Scientific or clinical background	☑	☐	☐	
	5	Rationale for using a radiomic approach	☑	☐	☐	
	6	Study objective(s)	☑	☐	☐	
**Method**						
Study Design	7	Adherence to guidelines or checklists (e.g., CLEAR checklist)	☑	☐	☐	
	8	Ethical details (e.g., approval, consent, data protection)	☑	☐	☐	
	9	Sample size calculation	☑	☐	☐	
	10	Study nature (e.g., retrospective, prospective)	☑	☐	☐	
	11	Eligibility criteria	☑	☐	☐	
	12	Flowchart for technical pipeline	☑	☐	☐	
Data	13	Data source (e.g., private, public)	☑	☐	☐	
	14	Data overlap	☑	☐	☐	
	15	Data split methodology	☑	☐	☐	
	16	Imaging protocol (i.e., image acquisition and processing)	☑	☐	☐	
	17	Definition of non-radiomic predictor variables	☑	☐	☐	
	18	Definition of the reference standard (i.e., outcome variable)	☑	☐	☐	
Segmentation	19	Segmentation strategy	☑	☐	☐	
	20	Details of operators performing segmentation	☑	☐	☐	
Pre-processing	21	Image pre-processing details	☑	☐	☐	
	22	Resampling method and its parameters	☑	☐	☐	
	23	Discretization method and its parameters	☑	☐	☐	
	24	Image types (e.g., original, filtered, transformed)	☑	☐	☐	
Feature extraction	25	Feature extraction method	☑	☐	☐	
	26	Feature classes	☑	☐	☐	
	27	Number of features	☑	☐	☐	
	28	Default configuration statement for remaining parameters	☑	☐	☐	
Data preparation	29	Handling of missing data	☑	☐	☐	
	30	Details of class imbalance	☑	☐	☐	
	31	Details of segmentation reliability analysis	☑	☐	☐	
	32	Feature scaling details (e.g., normalization, standardization)	☑	☐	☐	
	33	Dimension reduction details	☑	☐	☐	
Modeling	34	Algorithm details	☑	☐	☐	
	35	Training and tuning details	☑	☐	☐	
	36	Handling of confounders	☑	☐	☐	

	37	Model selection strategy	☑	☐	☐	
Evaluation	38	Testing technique (e.g., internal, external)	☑	☐	☐	
	39	Performance metrics and rationale for choosing	☑	☐	☐	
	40	Uncertainty evaluation and measures (e.g., confidence intervals)	☑	☐	☐	
	41	Statistical performance comparison (e.g., DeLong's test)	☑	☐	☐	
	42	Comparison with non-radiomic and combined methods	☑	☐	☐	
	43	Interpretability and explainability methods	☑	☐	☐	
**Results**						
	44	Baseline demographic and clinical characteristics	☑	☐	☐	
	45	Flowchart for eligibility criteria	☑	☐	☐	
	46	Feature statistics (e.g., reproducibility, feature selection)	☑	☐	☐	
	47	Model performance evaluation	☑	☐	☐	
	48	Comparison with non-radiomic and combined approaches	☐	☑	☐	
**Discussion**						
	49	Overview of important findings	☑	☐	☐	
	50	Previous works with differences from the current study	☑	☐	☐	
	51	Practical implications	☑	☐	☐	
	52	Strengths and limitations (e.g., bias and generalizability issues)	☑	☐	☐	
**Open Science**						
Data availability	53	Sharing images along with segmentation data [n/e]	☐	☑	☐	
	54	Sharing radiomic feature data	☑	☐	☐	
Code availability	55	Sharing pre-processing scripts or settings	☑	☐	☐	
	56	Sharing source code for modeling	☑	☐	☐	
Model availability	57	Sharing final model files	☑	☐	☐	
	58	Sharing a ready-to-use system [n/e]	☑	☐	☐	

Yes, details provided; **No**, details not provided; **n/e**, not essential; **n/a**, not applicable

Note: Use the checklist in conjunction with the main text for clarification of all items. Fill the "Page" column with the related page number where the information is provided.

Radiomics Quality Score(RQS) Checklist

Image protocol quality – well-documented image protocols (for example, contrast, slice thickness, energy, etc.) and/or usage of public image protocols allow reproducibility/replicability

- [x] protocols well documented
- [ ] public protocol used
- [ ] none

Multiple segmentations – possible actions are: segmentation by different physicians/algorithms/software, perturbing segmentations by (random) noise, segmentation at different breathing cycles. Analyse feature robustness to segmentation variabilities

- (●) yes
- ( ) no

Phantom study on all scanners – detect inter-scanner differences and vendor-dependent features. Analyse feature robustness to these sources of variability

- (●) yes
- ( ) no

Imaging at multiple time points – collect images of individuals at additional time points. Analyse feature robustness to temporal variabilities (for example, organ movement, organ expansion/shrinkage)

- (●) yes
- ( ) no

Feature reduction or adjustment for multiple testing – decreases the risk of overfitting. Overfitting is inevitable if the number of features exceeds the number of samples. Consider feature robustness when selecting features

- (●) Either measure is implemented
- ( ) Neither measure is implemented

Multivariable analysis with non radiomics features (for example, EGFR mutation) – is expected to provide a more holistic model. Permits correlating/inferencing between radiomics and non radiomics features

- ( ) yes
- (●) no

Detect and discuss biological correlates – demonstration of phenotypic differences (possibly associated with underlying gene—protein expression patterns) deepens understanding of radiomics and biology

- ( ) yes
- (●) no

Cut-off analyses – determine risk groups by either the median, a previously published cut-off or report a continuous risk variable. Reduces the risk of reporting overly optimistic results

- ( ) yes

- [x] no

Discrimination statistics – report discrimination statistics (for example, C–statistic, ROC curve, AUC) and their statistical significance (for example, p–values, confidence intervals). One can also apply resampling method (for example, bootstrapping, cross–validation)

- [x] a discrimination statistic and its statistical significance are reported
- [x] a resampling method technique is also applied
- [ ] none

Calibration statistics – report calibration statistics (for example, Calibration–in–the–large/slope, calibration plots) and their statistical significance (for example, P–values, confidence intervals). One can also apply resampling method (for example, bootstrapping, cross–validation)

- [x] a calibration statistic and its statistical significance are reported
- [x] a resampling method technique is applied
- [ ] none

Prospective study registered in a trial database – provides the highest level of evidence supporting the clinical validity and usefulness of the radiomics biomarker

- [ ] yes
- [x] no

Validation – the validation is performed without retraining and without adaptation of the cut–off value, provides crucial information with regard to credible clinical performance

- [ ] No validation
- [x] validation is based on a dataset from the same institute
- [ ] validation is based on a dataset from another institute
- [ ] validation is based on two datasets from two distinct institutes
- [ ] the study validates a previously published signature
- [ ] validation is based on three or more datasets from distinct institutes

Comparison to 'gold standard' – assess the extent to which the model agrees with/is superior to the current 'gold standard' method (for example, TNM–staging for survival prediction). This comparison shows the added value of radiomics

- [x] yes
- [ ] no

Potential clinical utility – report on the current and potential application of the model in a clinical setting (for example, decision curve analysis).

- [x] yes
- [ ] no

Cost–effectiveness analysis – report on the cost–effectiveness of the clinical application (for example, QALYs generated)

- ○ yes
- ◉ no

Open science and data – make code and data publicly available. Open science facilitates knowledge transfer and reproducibility of the study

- ☐ scans are open source
- ☑ region of interest segmentations are open source
- ☐ the code is open sourced
- ☑ radiomics features are calculated on a set of representative ROIs and the calculated features and representative ROIs are open source

Total score

19
(52.78%)

Statement of Informed Consent

Statement of Informed Consent

The Ethical Review Committee of National Institute of Parasitic Diseases, Chinese Center for Disease Control and Prevention has reviewed this project, The ethics review number is: 2021019.

We hereby affirm that our research plan has been executed in strict adherence to the ethical principles outlined in the Helsinki Declaration. Institutional review and approval were granted by the National Institute of Parasitic Diseases, Chinese Center for Disease Control and Prevention. Prior to their participation, all subjects involved in the study were comprehensively informed about the research aims, procedures, potential benefits, and risks. We have obtained written informed consent from every participant, ensuring that they have voluntarily agreed to take part in this research with a full understanding of their role.

NATIONAL INSTITUTE OF PARASITIC DISEASES
CHINESE CENTER FOR DISEASE CONTROL AND PREVENTION

CHINESE CENTER
FOR TROPICAL DISEASES RESEARCH

NATIONAL CENTER FOR INTERNATIONAL
RESEARCH ON TROPICAL DISEASES

中国疾病预防控制中心寄生虫病预防控制所

（国家热带病研究中心）

伦 理 审 查 委 员 会

THE NATIONAL INSTITUTE OF PARASITIC DISEASES (NIPD),

CHINESE CENTER FOR DISEASE CONTROL AND PREVENTION

（CHINESE CENTER FOR TROPICAL DISEASES RESEARCH）

ETHICAL REVIEW COMMITTEE

Approval Notice

PRINCIPAL INVESTIGATOR OF PROJECT: Zhou Xiaonong

TITLE OF PROJECT: Research on Pathogenic Mechanism and Prevention and Control Intervention Technology of Important Threating Human Parasites （2021YFC2300800）

INSTITUTE: National Institute of Parasitic Diseases, China CDC

Number: 2021019

The Ethical Review Committee of National Institute of Parasitic Diseases, Chinese Center for Disease Control and Prevention has reviewed the proposal of "Research on Pathogenic Mechanism and Prevention and Control Intervention Technology of Important Threating Human Parasites, Topic 1: Mechanisms of pathogenic and immunosuppressive function of important parasite on host, Topic 2: Mechanisms of cross-species transmission of important parasites, Topic 3: Research on the molecular basis of co-evolution of important parasites and hosts, and Topic 4: Establishment of emulator and prediction modelling for important parasitic diseases and empirical investigation on precision intervention by 'One health' approach". This project only deals with collecting elementary information about social, environmental, household and individual characteristics, collecting biological materials including blood, marrow, cerebrospinal fluid and feces samples of human beings. It is recognized that all those methods for survey and samples collecting will be well accepted by the local people, the right and the welfare of the subject are adequately protected and the potential risks are outweighed by potential benefits.

SIGNATURE: Cao Jianping

Vice Chair , Ethical Review Committee, NIPD, China CDC

Dec 20, 2021

Reference

1. Kocak, B., Akinci D'Antonoli, T., Mercaldo, N., Alberich-Bayarri, A., Baessler, B., Ambrosini, I, et al. (2024). METhodological RadiomICs Score (METRICS): a quality scoring tool for radiomics research endorsed by EuSoMII. Insights into imaging, 15(1), 8.
